# Improving Gender and Sexuality Inclusivity of a Long-Running HIV Behavioural Surveillance Survey to Identify New Sexual Practices, HIV Risks, and Maintain Community Support: A Mixed Methods Study

**DOI:** 10.1007/s10461-025-05006-0

**Published:** 2025-12-23

**Authors:** Anthony K. J. Smith, Christy E. Newman, Benjamin R. Bavinton, Curtis Chan, Timothy R. Broady, James MacGibbon, Daniel Vujcich, Tina Gordon, James Gray, Martin Holt

**Affiliations:** 1 Centre for Social Research in Health, UNSW Sydney, Sydney, Australia; 2 Australian Human Rights Institute, UNSW Sydney, Sydney, Australia; 3 School of Population Health, Curtin University, Perth, Australia; 4 Collaboration for Evidence, Research and Impact in Public Health, Curtin University, Perth, Australia; 5 The Kirby Institute, UNSW Sydney, Sydney, Australia; 6 WAAC, Perth, Australia; 7 NSW Ministry of Health, Sydney, Australia; 8 Health Equity Matters, Sydney, Australia

**Keywords:** Australia, Cognitive interviews, Gay and bisexual men, Non-binary, Survey design, Trans and gender diverse

## Abstract

Inclusive language in data collection is essential for effective and sustained engagement with marginalised populations. The historic use of imprecise conceptualisations of gender and sexuality in the field of HIV and sexual health research has been challenged in the last decade, particularly cisnormative and monosexist assumptions about identity and practice. This study reports on the process of redesigning a repeated cross-sectional survey questionnaire in Australia to improve gender and sexuality inclusivity. The Gay Community Periodic Surveys (GCPS), repeated since 1996, was redesigned in 2023 to be more inclusive of transgender men, non-binary people, and bi + participants. Changes included a title change (GBQ + Community Periodic Surveys), adoption of gender-neutral language, a shift from an exclusive focus on anal sex, enhanced questions about sex with female partners, new questions about non-binary partners, and better recognition of non-monogamous relationships. Mixed methods were employed to inform and evaluate these changes, including cognitive interviews (*n* = 30) with a diverse range of gay, bi+, and queer men (cis and trans) and non-binary people, and rapid field interviews (*n* = 39) with participants who had completed the new questionnaire. Thematic analysis showed that the changes were acceptable, but the maintenance of some cisnormative assumptions about bodies and practices were also observed. The questionnaire will continue to be adapted to recognise a broader range of practices and risks within new subgroups of gay, bi, and queer men and non-binary people, with sensitivity to community expectations about survey length and comprehensibility of terms.

## Resumen

El lenguaje inclusivo en la recopilación de datos es esencial para una participación efectiva y continua con las poblaciones marginadas. En las últimas décadas se han cuestionado conceptualizaciones imprecisas sobre el género y la sexualidad en el campo de la investigación sobre VIH y salud sexual, particularmente los supuestos cisnormativos y monosexistas usados para describir la identidad y las prácticas. Este estudio presenta el proceso de rediseño de un cuestionario de encuesta transversal aplicada de manera periódica en Australia para mejorar la inclusión de género y sexualidad. Las Encuestas Periódicas de la Comunidad Gay (GCPS), realizadas desde 1996, se rediseñaron en 2023 para ser más inclusivas con las personas transgénero, no binaries y los participantes bi+. Los cambios incluyeron un cambio de título (Encuestas Periódicas de la Comunidad GBQ+), la adopción de un lenguaje neutral en cuanto al género, un cambio de perspectiva que incluya otras prácticas además del sexo anal, preguntas más sensibles sobre el sexo con parejas femeninas, nuevas preguntas sobre parejas no binaries y un mejor reconocimiento de las relaciones no monógamas. Se emplearon métodos mixtos para orientar y evaluar estos cambios, incluyendo entrevistas cognitivas (*n* = 30) con una gama diversa de personas gay, bi + y queer (cisgénero y transgénero) y no binaries, y entrevistas rápidas de campo (*n* = 39) con participantes que completaron el nuevo cuestionario. El análisis temático mostró que los cambios eran aceptables, pero también se observó que mantenían algunos supuestos cisnormativos sobre cuerpos y prácticas. El cuestionario continuará adaptándose para reconocer una gama más amplia de prácticas y riesgos dentro de los nuevos subgrupos de personas gay, bi + y queer, y no binaries, teniendo en cuenta las expectativas de la comunidad sobre la extensión de la encuesta y la comprensión de los términos.

## Introduction

Since the emergence of the HIV pandemic, behavioural surveillance has been key to understanding and monitoring a range of HIV and sexual health-related practices, including numbers of sexual partners, HIV and sexually transmissible infection (STI) testing, HIV prevention, treatment, and patterns of drug use. International guidelines have emphasised the value of behavioural surveillance to track and monitor the health and drivers of HIV/STI infection with affected populations [1, 2]. In many countries, gay, bisexual, and other men who have sex with men (GBMSM) are disproportionately affected by HIV and STIs, and a variety of surveillance systems have been used to assess their HIV-related behaviour and health needs [3], although few of these surveys are regularly conducted [4]. 

In settings where GBMSM are a key population for HIV prevention, the majority of behavioural surveillance has focused on cisgender gay men, and advocates and researchers within the last decade have identified how the experiences of gender and sexuality diverse subpopulations of GBMSM have often been neglected [5–8]. HIV studies have tended to either assume participants are cisgender (cis), failed to ask appropriate questions to identify transgender (trans) and gender diverse (TGD) participants from cis participants, conflated men who have sex with men (MSM) with trans women, and excluded trans men from studies focused on men [7, 8]. While bisexual men have participated in many GBMSM studies, research and surveillance have historically neglected understanding the distinct identities and experiences of bi + people (bisexual, pansexual, and other people with multi-gender attraction), for example by not assessing their identities and relationships beyond sex with men [6]. These practices have been described as contributing to the social ‘erasure’ of bisexual and TGD experience [6–8]. 

There are numerous reasons for the lack of visibility or focus on bisexual and TGD experiences in HIV and sexual health behavioural surveillance: it is the outcome of a focus on epidemiological risk (focusing on sex between men); a legacy of twentieth century understandings of gender and sexuality; researchers’ normative assumptions about gender and bodies being reproduced in study design; a presumption that only cisgender people will participate; and a consequence of omitting questions that enable participants’ demographic details (particularly gender and sexual orientation) to be accurately captured. Focusing HIV and sexual health behavioural surveillance on populations with the highest risk of HIV is understandable given the need to prioritise funding. However, this epidemiological profile is changing, and so too are understandings of gender and sexuality and patterns of sexual relationships, including in ways that trouble the stability of the category of ‘MSM’ [5, 8–10]. In Australia, where this study is based, there has been a 33% decline in HIV notifications between 2014 and 2023 (722 diagnoses in 2023; 63% male-to-male sex), driven primarily by a decrease amongst Australian-born MSM [11]. Australia’s current national HIV strategy (2024–2030) acknowledges that research on HIV-related behaviour among bisexual men and TGD people is lacking [12]. Australia’s national epidemiological reporting on HIV cases still does not report sexual orientation, continuing to focus on mode of transmission (therefore making it difficult to identify bi + men) and inadequately categorises TGD people [11]. As Australia’s HIV epidemic becomes more concentrated, it is vital to improve behavioural surveillance to be more nuanced in how TGD people are recorded (including within GBMSM studies), and to be able to disaggregate the specific experiences of bi + participants from other MSM [5, 6, 13]. 

Gender and sexuality inclusive research practices have improved in some contexts by deliberately developing guidelines on the appropriate collection of information on sex, gender, and sexual orientation [13, 14]. However, there remain many challenges in achieving meaningful inclusion of gender and sexuality diversity in research. While a focus on epidemiological risk (e.g., sex between men) may be warranted, language and concepts of sex, gender, and bodies have changed, and not keeping pace with community understandings risks inaccurate science and poor community acceptability. For example, how non-binary people fit into health services and research studies with largely binary gendered systems must be meaningfully addressed, especially as a growing number of young people identify outside of binary gender categories in high-income English-speaking countries [15–18]. In the present study, a growing number of non-binary people who have sex with men have participated in regular surveys since introducing expanded gender options from 2017 (e.g., shifting from 1.3% non-binary people in 2018 to 4.2% in 2023 in Melbourne [19, 20]), and their epidemiological risk was similar to participants identifying as men.

Ensuring the collection of accurate data through epidemiology and behavioural surveillance is vital for effective responses to HIV [1, 2]. Inclusive research practices, including the use of community relevant language and terminology that reflects local norms about gender and sexuality, are vital to showing respect to participants, improve recruitment, and ensure accurate results [16, 21, 22]. In the context of survey questionnaires, poorly worded questions can increase response burden and result in incomplete participation or poor-quality data if participants are unable to comprehend the meaning of the questions [23–25]. Further, questionnaires that embed heteronormative, cisnormative, and monosexist assumptions may also reduce the willingness of bi + and TGD people to participate. Our understanding of and the language used to express gender and sexuality are continuously transforming [10]. A key challenge for those involved in leading longitudinal and repeated studies – which provide essential insights into the changing practices and needs of HIV-affected communities – is adapting survey instruments to align with changes in community understandings of gender and sexuality [3, 16]. This paper reports on the process of seeking to make a longstanding repeated survey for GBMSM more gender and sexuality inclusive.

## The Questionnaire

Since 1996, the Gay Community Periodic Surveys (GCPS) have formed a key part of Australia’s behavioural surveillance system for HIV, sexual health, and drug use [26, 27]. The questionnaires were designed in partnership between researchers, community organisations and government policymakers to be relevant for community members and produce data that could shape education, prevention, and health promotion programs. They are regularly cited in national and state HIV strategies, and are used to monitor National HIV Strategy targets, including the level of HIV prevention coverage amongst GBMSM [12]. The GCPS involve repeated, cross-sectional surveys of GBMSM in seven out of eight Australian states and territories. The research team collaborates with state- and territory-based LGBTQ + community health organisations, who train and hire peer recruiters to distribute the survey face to face during LGBTQ + festival periods (e.g. Sydney Gay and Lesbian Mardi Gras, Melbourne’s Midsumma Festival). This event- and venue-based recruitment is supplemented by online advertising and an online version of the questionnaire to maximise participation.

The questionnaire and recruitment approaches have been adapted over time to the changing context of HIV and the ways that GBMSM socialise, including introducing online recruitment, adjusting the survey questionnaire in response to COVID-19, and adding questions about PrEP and mpox [28–30]. This is a key part of conducting behavioural surveillance; refining and adjusting the survey instrument to anticipate and follow changes in community practice and emerging health threats [1–3, 27]. 

Since 2018 there has been an increase in feedback that was critical of the GCPS from bi + and trans communities. This feedback was given by participants to recruitment staff, raised by community organisations and their staff, or sent directly by participants to the research team. The feedback noted that it was unclear if non-binary participants were eligible to participate, that the title and framing of questions was not inclusive of bi + participants, and that the survey did not ask sufficient questions about female or non-binary partners. In 2017, following feedback from community partners and trans men, the GCPS introduced separate gender and sex questions to be able to identify cis, trans and non-binary participants. However, in some parts the questionnaire still presumed cis male respondents (e.g., it only asked about anal sex, and did not ask about vaginal or fronthole swabs for STI testing), and the eligibility of non-binary people remained ambiguous, even though the number of non-binary people participating in the survey was increasing over time [19, 20]. 

This paper reports on a sub-study that was designed to improve the gender and sexuality inclusivity of the GCPS questionnaire by formally engaging with community members to consider both the existing questions and proposed changes. Along with improving the survey’s inclusivity for bi + and TGD participants, we also made sure that any changes to the questionnaire were clear and appropriate for cis gay men, ensuring the revisions were relevant for all groups.

## Methods

### Questionnaire Redesign

In early 2023, the project team met to plan a preliminary redesign of the questionnaire. An initial draft was shared with the national GCPS advisory group, including 22 organisations with expertise in LGBTQ + communities and HIV, state and territory health departments, community-based organisations, and other research institutions. This consultation confirmed broad support for the redesign and provided further suggestions for changes to the questionnaire. From this process, a revised version of the questionnaire was used for cognitive interviewing (described below) to test comprehension and solicit further feedback from potential participants. Following cognitive interviewing, further changes were made to the questionnaire and taken back to the advisory group for approval. The final revised version of the questionnaire was first used in 2024 and evaluated in the first location it was used through rapid field interviews (described below).

### Study Design

Two qualitative methods, cognitive interviewing and rapid field interviews, were employed to test and evaluate the comprehension and acceptability of the redesigned questionnaire. Cognitive interviewing involved a semi-structured interview format during which research participants were presented with the revised GCPS questionnaire and asked to complete it while discussing their experience of doing so, to investigate how questions were understood and whether they fulfilled their intended purposes [23, 24]. Rapid field interviews involved ‘vox pop’ interviews (lasting 1–2 min) during a daytime LGBTQ + festival event where in-person recruitment for the GCPS was taking place. Participants were approached after they had completed the questionnaire and asked for their impressions and feedback on the survey. All participants provided written or audio-recorded verbal consent prior to data collection. The study was approved by the UNSW Human Research Ethics Committee (HC230384) and the community research review panels of ACON (202323) and Thorne Harbour Health (23010).

### Recruitment and Sampling

Cognitive interview participants were recruited through unpaid social media advertisements (on Meta) and email networks, with assistance from community partner organisations. After clicking on an advertisement or link, potential participants were asked to complete an online expression of interest form to assist with ensuring a maximum variation sample of demographically diverse participants. Sample targets included ensuring recruitment of at least four trans men, non-binary people, people born overseas in non-English speaking countries, people living with HIV, young participants (18–29) and older participants (50 and above), and all targets were met. Eligible participants were aged 18 years or older, spoke English, lived in Australia, and were gay, bi, or queer men (cis or trans), men who had had sex with men in the past 5 years, or non-binary people who had had sex with gay, bi, or queer men in the past 5 years. On interview completion, participants were offered an AUD$50 gift card in recognition of their time.

Rapid field interviews were conducted in January 2024 during the outdoor Carnival event (part of the Midsumma festival in Melbourne). Convenience sampling was employed, and eligible participants were aged 18 years or older and had completed a GCPS questionnaire. No compensation was offered.

### Data Collection

Interview guides were developed by the study team. Cognitive interviewing was conducted by A.S. during August-October 2023 by videoconferencing or telephone, based on participant preference. Participants were asked about their personal background, any experiences they had had participating in the GCPS or similar research, and were then presented with the questionnaire (on screen or with a PDF provided by email). They were then asked to answer the questions and prompted to share their thoughts about the wording of questions and answer options. General probes, such as ‘why do you think these questions are asked?’, ‘how did you come up with your answer?’ were asked as they moved through the questionnaire [23], with specific probing based on some items, e.g., ‘do you know the gender of people you have sex with?’ At the end of the interview, participants were asked to reflect on the overall questionnaire, and to provide any other feedback to improve inclusivity and the ease of survey completion. Following interviews, demographic questions were asked (gender and sex assigned at birth, sexual orientation, age, state of residence, postcode, HIV status, Aboriginal or Torres Strait Islander origin, and cultural or ethnic background), and participants chose a pseudonym or asked the interviewer to choose one. Interviews lasted an average of 66 min (range 34 to 99 min). Fieldnotes were written about each interview, summarising feedback on each survey item and the issues raised, and these notes informed the process of coding and analysis.

Field interviews were conducted in person, and due to the public setting, only involved asking basic non-intrusive demographic questions (age, gender identity, and sexual orientation), followed by asking how participants felt about completing the survey, whether it was their first time participating, why they had decided to do the survey, what they thought its purpose was, and anything they would change about the questionnaire. Field interview participants were assigned a code rather than a pseudonym. Interviews lasted an average of 2 min (range 1 to 5 min). An overall set of fieldnotes were written at the end of the day to reflect on data collection.

All interviews were audio-recorded, professionally transcribed verbatim, and checked for accuracy. Transcripts were deidentified to ensure all people, places, and organisation names were replaced or removed.

### Data Analysis

Analysis of the cognitive interviews focused on identifying problems with comprehension, item vagueness, and potential solutions, [23] and analysis of both sets of interviews focused on understanding the perception, acceptability, and inclusivity of the GCPS. Thematic analysis [31] was conducted through familiarisation with the deidentified transcripts by A.S., writing memos to reflect on emerging insights, and line by line coding of relevant extracts. The process of analysis and developing themes involved regular discussions with the study team. This analysis centred on the acceptability of the questionnaire, items that required additional work, and participants’ views about the detail that could or should be identified in questions about sexual practices.

## Results

Thirty cognitive interviews were completed. Table 1 contains a summary of participant demographics. Most participants identified as cis men (63.3%), nearly half as gay (46.7%) with varying proportions of bi + and queer participants; and most were aged in their 30 s (53.3%). More than half the participants were born in Australia (63.3%), with eleven of those born in Australia reporting an Anglo or white ethnic background. More than half the participants reported being HIV-negative and not using PrEP (56.7%), a quarter were HIV-negative and using PrEP (26.7%), and five participants were living with HIV (16.7%). Most interviews were conducted via video conferencing, with two by telephone.

**Table 1:** Cognitive interview participant demographics

Demographic variable	Total, *n*=30
Gender	
Cis men	19
Non-binary	7
Trans men	4
Sexual orientation	
Gay	14
Bi, bisexual and/or pansexual	7
Queer	6
Queer/bisexual	3
Age	Mean = 37
20s	5
30s	16
40s	4
50s	3
60s	2
State	
New South Wales	22
Victoria	4
Australian capital territory	3
Queensland	1
HIV status and ARV use	
Living with HIV, undetectable viral load	5
HIV-negative, not using PrEP	17
HIV-negative, daily PrEP	6
HIV-negative, on-demand PrEP	2
Country of Birth	
*Australia	19
Aotearoa New Zealand	3
Southern and Central Asia	3
South-East Asia	2
Eastern Europe	1
North-East Asia	1
United Kingdom	1

*Amongst those born in Australia, 8 reported non-Anglo or white ethnic backgrounds, including from Europe, the Middle East, South America, Central Asia, and South-East Asia

No participants identified as Aboriginal or Torres Strait Islander

A total of 39 participants took part in field interviews. Table 2 contains a summary of the limited participant demographics that were requested. Most participants identified as men (79.5%), most as gay (71.8%), and half were aged in their 30 s (53.8%).

**Table 2:** Field interview participant demographics

Demographic variable	Total, *n*=39
Gender*	
Male or man	26
Cisgender male or man	4
Non-binary	4
Trans woman	2
Trans man	1
Genderfluid	1
Unsure	1
Sexual orientation	
Gay or homosexual	28
Straight or heterosexual	3
Bisexual, pan, or pansexual	3
Queer	3
Queer, gay, and lesbian	1
No answer	1
Age	Mean = 37.8
18–19	1
20s	6
30s	21
40s	4
50s	5
60s	1
70s	1

### Questionnaire Revisions

Table 3 contains a summary of key changes across three different versions of key questions: the original questionnaire, the redesigned version tested in cognitive interviews, and the final version evaluated through rapid field interviews. Full versions are publicly available as appendices, including the original questionnaire [20] and the final version [32]. In summary, the questionnaire title was changed from the ‘Gay Community Periodic Survey’ to the ‘GBQ + Community Periodic Survey’, retaining the ‘GCPS’ acronym and branding. Gender-neutral language was added throughout (not assuming the participant or their partners were all men, for example), and sexual practice questions were refined to no longer limit the definition of intercourse to anal sex. New sections asking specifically about sex with female and non-binary partners were also added.

**Table 3:** Summary of key questionnaire changes

Questionnaire item	Initial questionnaire	Cognitive interviewing questionnaire	Field questionnaire
Title	Gay Community Periodic Survey	GBQ+ Community Periodic Survey	Unchanged from cognitive interviewing
Community connectedness	Q: How many of your friends are gay men? A: None, a few, some, most, all Q: How much of your free time is spent with gay men? A: None, a little, some, a lot	Q: How many of your friends are *gay*,* bi or queer people*? A: None, a few, some, most, all Q: How much of your free time is spent with *gay*,* bi or queer people*? A: None, a little, some, a lot	Unchanged from cognitive interviewing
Gender	Q: What is your gender? A: Male, Woman, Non-binary, Other	Q: What is your gender? A: Male, Woman, Non-binary, Other	Q: What is your gender? A: Man, Woman, Non-binary, *Another term [open field]*
Sex recorded at birth	Q: What sex was recorded on your birth certificate? A: Male, Female, Other	Q: What sex was recorded on your birth certificate? A: Male, Female, *Another Term*	Unchanged from cognitive interviewing
Sexual orientation	Q: Do you think of yourself as: A: Gay, Bisexual or pansexual, Heterosexual, Queer, Other [open field]	Unchanged from initial questionnaire	Q: What is your sexuality? A: Gay, Bisexual or pansexual, Heterosexual, Queer, *Another term* [open field]
Numbers of sexual partners (men)	Q: How many different men have you had with in the last 6 months? Include cis and trans men. A: None, One, 2–5 men, 6–10 men, 11–20 men, 21–50 men, more than 50 men	Q: How many different men have you had sex with in the last 6 months? Include cis and trans men. A: None, One *man*, 2–5 men, 6–10 men, 11–20 men, *>20 men*	Unchanged from cognitive interviewing
Number of sexual partners (women)	Q: How many different women have you had with in the last 6 months? Include cis and trans women. A: None, One, 2–5 women, 6–10 women, more than 10 women	Q: How many different women have you had sex with in the last 6 months? Include cis and trans women. A: None, One *woman*, 2–5 women, 6–10 women, 11–20 women, *>20 women*	Unchanged from cognitive interviewing
Number of sexual partners (non-binary)	This question did not exist in this version.	Q: How many different non-binary people have you had sex with in the last 6 months? A: None, One person, 2–5 people, 6–10 people, 11–20 people, >20 people	Unchanged from cognitive interviewing
Condom use with regular male partners Note - these questions are repeated for casual partners, replacing the word ‘regular’ with ‘casual’.	Q: Have you had sex with a regular male partner/s in the last 6 months? A: Yes, No [If yes] Anal sex regular partner/s: Q: I fucked him with a condom. A: Never, Occasionally, Often Q: He fucked me with a condom. A: Never, Occasionally, Often Q: I fucked him without a condom A: Never, Occasionally, Often Q: He fucked me without a condom A: Never, Occasionally, Often	Q: Have you had sex with a regular male partner *or partners* in the last 6 months? *Include cis and trans men.* A: Yes, No [If yes] Q: *When you fucked or were fucked by your regular male partner(s)*,* how often did you use condoms?* A: *Never*,* Occasionally*,* Often*,* Always*,* No Fucking*	Unchanged from cognitive interviewing
Condom use with regular female partners Note - these questions are repeated for casual partners, replacing the word ‘regular’ with ‘casual’.	This question did not exist in this version.	*Q: Have you had sex with a regular female partner or partners in the last 6 months? Include cis and trans women.* *A: Yes*,* No* *[If yes] Q: When you fucked or were fucked by your regular female partner(s)*,* how often did you use condoms?* *A: Never*,* Occasionally*,* Often*,* Always*,* No Fucking*	Unchanged from cognitive interviewing
Condom use with regular non-binary partners Note - these questions are repeated for casual partners, replacing the word ‘regular’ with ‘casual’.	This question did not exist in this version.	*Q: Have you had sex with a regular non-binary partner or partners in the last 6 months?* *A: Yes*,* No* *[If yes] Q: When you fucked or were fucked by your regular non-binary partner(s)*,* how often did you use condoms?* *A: Never*,* Occasionally*,* Often*,* Always*,* No Fucking*	Unchanged from cognitive interviewing
Sexual health tests	Q: Which of these sexual health tests have you had in the last 12 months? A: Anal swab Throat swab Urine sample Blood test for syphilis Other blood test (answer options none, one, two, 3, 4+ for each)	Q: Which of these sexual health tests have you had in the last 12 months? A: Anal swab Throat swab *Vaginal/fronthole swab* Urine sample Blood test for syphilis Other blood test (answer options none, one, two, *3+* for each)	Q: Which of these sexual health tests have you had in the last 12 months? A: Anal swab Throat swab Vaginal *or* fronthole swab Urine sample Blood test for syphilis (answer options none, one, two, 3+ for each)
Relationship status and structure	Q: Are you currently in a relationship with a man? e.g. a boyfriend, long term partner or husband, cis or trans A: Yes, No [section ends] Q: How would you describe your relationship? (choose one) A: We are monogamous – neither of us has casual sex We have an open relationship – my partner or I have casual sex I am in a relationship with several men	Q: Are you currently in a relationship? A: Yes, No [section ends] *Q: Who are you in a relationship with? Tick all that apply* *A: Man*,* Woman*,* Non-binary person*,* More than one person* Q: How would you describe your relationship? (choose one) A: We are monogamous – neither of us has casual sex We have an open relationship – my partner or I have casual sex I am in a relationship with several *people*	Q: Are you currently in a relationship? A: Yes, No [section ends] Q: Who are you in a relationship with? Tick all that apply A: Man, Woman, Non-binary person, More than one person Q: How would you describe your relationship*(s)*? (choose one) A: We are monogamous or closed – *we don’t* have casual sex We are open – my partner*(s)* or I have casual sex
Relationship agreement	Q: Do you have a clear (spoken) agreement about sex within your relationship? A: No agreement Agreement: No sex Agreement: No anal sex Agreement: All anal sex is with a condom, Agreement: Anal sex can be without a condom Q: Do you have a clear (spoken) agreement about sex with casual male partners? A: No agreement Agreement: No sex Agreement: No anal sex Agreement: All anal sex is with a condom, Agreement: Anal sex can be without a condom	Q: Do you have a clear (spoken) agreement about sex within your relationship? A: No agreement, Agreement: No sex, Agreement: No *fucking e.g. oral sex only*, Agreement: All *fucking* is with a condom, Agreement: *Fucking* can be without a condom Q: Do you have a clear (spoken) agreement about sex with casual partners? A: No agreement Agreement: No *casual* sex *allowed* Agreement: No *fucking with casual partners* Agreement: All *fucking* is with a condom Agreement: *Fucking* can be without a condom	Q: *With your relationship partner(s)*, do you have a clear (spoken) agreement about the sex *you have together*? A: No agreement Agreement: No sex Agreement: No fucking e.g. oral sex only Agreement: All fucking is with a condom Agreement: Fucking can be without a condom *Q: With your relationship partner(s)*, do you have a clear (spoken) agreement about sex with casual partners? A: No agreement Agreement: No casual sex allowed Agreement: No fucking with casual partners Agreement: All fucking is with a condom Agreement: Fucking can be without a condom

*Q* Question, *A* answer options; wording changes in italics

Almost all cognitive interviewing participants indicated that the revised questionnaire was acceptable. Some of those who had completed it over many years observed a trend towards more inclusivity over time, meaning they could now properly ‘count’ their sexual partners:

I always write [feedback about the questionnaire] in the margins. The last one was ‘How can I articulate my non-binary partners?’ And now I can. It’s beautiful. I’ve seen incremental change every time for a decade, which is really cool to see. (Sam, queer/bisexual trans man).

The process of cognitive interviewing affirmed that the survey was easy to answer in most parts, and that the language was viewed as acceptable and relevant (e.g., ‘fucking’ was most often seen as appropriate, community-based language and well understood to refer to penetrative sex). The question of why the survey was being changed, and who would determine the success of those changes, was often implicit in responses. For example, Ben, a gay cis man, the original target population for this survey historically, shared that he believed the new version was “inclusive enough that I think you are going to satisfy the majority of people.”

The cognitive interviews contributed feedback that led to further improvements, including minor tweaks to readability and inclusivity. Some changes were simple; participants suggested ‘another term’ as a more inclusive answer option to ‘other’ (which was seen as a minimising or invalidating word by some). Trans men and non-binary participants also indicated a preference to be able to write in a specific option for gender, and to change ‘other’ to ‘another term’ throughout. As Dani (non-binary queer person) explained, “People don’t like to be ‘Other’. It’s othering, literally. […] I think we should allow people to specify, if it’s possible.”

Given that the questionnaire was asking about non-binary partners for the first time, we wanted to understand what ‘non-binary’ meant to participants and how they might determine and categorise the gender of their partners across the three gender categories being offered (men, women, non-binary people). All participants understood non-binary appropriately, but there were some differences in approaches to categorising partners based on whether participants had met sexual partners anonymously, casually, or by dating. For example, Ken explained, “The thing is I don’t know if any of them were non-binary because, yeah, I could very well have had sex with non-binary people. I don’t tend to ask the gender of my partners.” (Ken, bi cis man). Ken therefore categorised the gender of his partners based on assumptions. In contrast, other participants explained that they could confidently report the gender of their partners accurately. In all cases, the questions were well comprehended, and participants were able to answer the questions, but participants varied in their confidence about knowing and reporting their sexual partners’ genders.

Another insight gained from the cognitive interviews was that several participants felt the relationship section could be difficult to answer for people in relationships with multiple people, and that the wording used in this section was more difficult to comprehend than others. The two ‘relationship agreement’ items had notably poor comprehensibility, with many participants often misinterpreting the first question, assuming it was asking about sex with casual partners. When they moved to the second question they realised that the first question was actually asking about sex with relationship partners. Taking on this feedback, the questions were rephrased to improve comprehension, specifying that the agreements were about “the sex you have together” and “sex with casual partners”.

### Tensions over the Detail of Sex and Body Parts

A key tension raised through the cognitive interviewing was that while the survey used inclusive language, some trans participants were concerned that the research team would still have to make assumptions about the particular body parts and sexual acts that participants were reporting. For example, it was explained that the items relating to ‘condom use for partners’, particularly the question ‘when you fucked or were fucked by your regular/casual female partner(s), how often did you use condoms?’ presumed certain types of sex. As Amos explained:

It seemed to make assumptions about things. Like I don’t have straight sex with women, which would look more like penetrative, maybe with a strap-on or a cis-penis, but the questions about the female partners didn’t capture that a lot of non-binary people are queer and don’t have that kind of sex, and a lot of queer men might not be having penetrative sex with women as well. (Amos, non-binary queer person)

A few participants were familiar with an approach from other questionnaires which involves asking more detailed questions about body parts and practices used during sex [16], which as some argued, would elicit more detailed assessments of HIV risk.

Similar tensions were observed in items about HIV risk reduction strategies during condomless sex, in which language such as ‘pull out’ or ‘pulled out’ was seen as implying that particular body parts were being used by both the participant and the relevant sexual partner(s). For example, during the cognitive interviewing, some answer options described risk reduction strategies in HIV prevention such as ‘strategic positioning’ and ‘withdrawal’: e.g. “When they fucked me, I made sure they pulled out before cumming because their HIV status was different or unknown to me.” The presence of this type of phrasings led Dani (a non-binary queer person) to reflect:

[The revised survey instrument] feels exciting to some extent, but then also like a bit of false hope. [laughs] Especially when they’re really looking at HIV risk, and only really interested in one particular kind of sex, which is disappointing. It’s like they include people who are outside the cis gay male box, but they’re not really thinking about their actual experiences of sex and how sex is interpreted.”

On the one hand, a few trans participants wanted more precise questions about body parts and practices. On the other hand, many participants noted that the length of the survey (four A4 pages, 10 min on average to complete) was appropriate and that the questions already felt very in-depth.

In the questions about the number of sexual partners, a few participants also felt it would be valuable to distinguish between cis and trans partners:

I do struggle that cis and trans men and cis and trans women are collapsed. It just doesn’t produce meaningful data in terms of risk and the kinds of sex people are having. (Sam, queer/bisexual trans man)

Sam also suggested separating questions about non-binary sexual partners based on sex assigned at birth (e.g., number of non-binary, assigned male at birth partners; and number of non-binary, assigned female at birth partners), although this approach was not supported by other participants and community consultations. As Vinh (a non-binary queer person) noted, the questionnaire handled the category of non-binary in an inclusive way, but it also could not capture all the nuance and diversity of non-binary people:

Non-binary people are all being kind of aggregated into one category when that category can mean a lot of different things, different experiences, different body types, different engagements with sex, and I think it’s good that we’re including it here, but it really is only like a surface level snapshot of what that might look like.

As Vinh articulated, the GCPS is a ‘snapshot’, and the questionnaire generates an aggregated summary of patterns of sexual and other HIV-related behaviour in the community, rather than generating individualised, in-depth detail about a person’s behaviour and HIV risk (that might be captured in a case study or detailed sexual history).

Although trans participants indicated that the questionnaire was overall acceptable and broadly inclusive, there were clearly tensions being expressed over its depth and length, and whether parts of the questionnaire assumed types of sex or bodies.

### Acceptability in the Field

Following the cognitive interviews, changes were made to the survey questionnaire (see Table 3, Field Questionnaire column, full version available as appendix [32]). Rapid field interviews were conducted to evaluate the finalised questionnaire, and this involved asking people who had completed the questionnaire during a round of data collection to provide general verbal feedback. Participants indicated broad acceptability for the survey:

It felt good. It’s very thorough. I remember doing this survey a year ago when I was single. Now I’m in a relationship. My answers were very different, like a lot of all one-line no’s, but yeah, the questions are very detailed and very inclusive. (F11, gay man, aged 32)

It was okay. It was easy to understand the questions. No ambiguity. And I felt comfortable answering the type of questions as well. (F03, gay male, aged 35)

Although participants were not informed that the questionnaire had been recently revised, some noticed changes from previous years, and provided positive feedback:

I think this year, it’s like really nicely inclusive and it included a lot of people who might call themselves like gay, queer or men who have sex with men and not identify with those labels, but they could still answer those questions. I still think there’s like a very large emphasis on HIV and what that represents and means in our community and it’s really good to see that they’re actually extending that to not just men, they’re extending it. (F32, homosexual cisgendered male, aged 33)

It’s always interesting and it’s fascinating to see how some of the emphases have changed over the years. There’s a lot more detail about the various different sex options, different relationship categories, different identities. (F17, gay male, aged 76)

Feedback from gender diverse participants also suggested that the survey was acceptable and inclusive:

I really appreciated that there was a non-binary option on both surveys, and that it asked, you know, have you had sex with men, women, non-binary people, how many times, group sex, like it felt inclusive. (F25, queer/gay/lesbian non-binary, aged 38)

“I think [the survey] covered a lot of different options, it was good. It covered relationships and sexual activity for different genders. […] I mean, English is not my first language, and even in that case, I could just read everything properly and follow all the instructions. So, it was really clear. (F20, unsure of gender, gay, aged 29)

Overall, the consensus was that the survey was clear, inclusive, and easy to complete. However, one issue emerged during field interviews. During recruitment for the GCPS questionnaire, participants for a cervical cancer questionnaire were also being recruited, and some of the peer recruiters (recruiting for both surveys) had approached prospective participants and described the GCPS as ‘the men’s survey’ and the cervical cancer survey as ‘the women’s survey’ (which was not the approach recommended by the survey team or local partner organisation). This led to a few gender diverse participants expressing frustration:

[It’d be good if they were] giving the person the option of which survey they want to complete. So, my sister was just handed a specific survey. I was handed something completely different. And they didn’t actually have the chance to confirm that. (F16, genderfluid queer, 33)

Other recruiters offered the two survey options in a less binary gender way, giving prospective participants the choice to consider whether one or both questionnaires were relevant to complete e.g. ‘This survey is about HIV and sexual health, this one is about cervical cancer.’ This occurrence was an important reminder that inclusive changes need to be consistent across both the questionnaire and recruitment processes, and that it can be particularly challenging to overcome all elements of the binary gendered framing that underpins most health research and policy.

## Discussion

All research studies should be considering how they are conceptualising gender and sexuality in consultation with key communities, and doing so on an ongoing basis [21, 22]. This study utilised consultations, cognitive interviews, and field interviews to guide and evaluate the redesign of a longstanding HIV behavioural surveillance survey to be more inclusive of trans men, bi + people, and non-binary people who have sex with men. The overall feedback from participants was that the changes were acceptable and readily understood, and the benefit of undertaking a formal cognitive interviewing process also enabled the identification of other improvements to the questionnaire. However, there remained some tensions in community views on the changes, particularly regarding gender and bodies, about the precision of the data collected, and whether the questionnaire implied cisnormative assumptions. As a research team we aimed to balance a number of competing concerns: community expectations and acceptability of language and terminology; the burden of filling out the survey; the need to maintain public health utility and to be able to identify higher or lower levels of HIV risk and ensure continuity of analysis with past survey rounds.

Given that TGD people have often been less visible in past HIV and sexual health research [5, 7, 8], and as bi + men’s sexual experiences beyond sex with men have also often been overlooked [6], it is particularly vital to consider how cisnormative and monosexual assumptions shape the design and conduct of surveillance and health systems. The outcome of undertaking this process is that the GCPS is now able to more accurately ask questions about a greater range of sexual partners (particularly women and non-binary people) to identify sexual practices and HIV and STI risks. Further, the survey is likely to enjoy enhanced community support from participants (as reflected in the cognitive and field interviews), thereby maintaining community engagement and reducing the burden on individual participants.

Cognitive interviewing was valuable for generating in-depth feedback on questionnaire comprehension and acceptability. Several points of feedback were adapted into the final version, including small answer option changes (e.g., ‘another term’ rather than ‘other’) and changes to the relationships section to more explicitly include people involved in multiple partner relationships.

Reviewing a section on risk reduction, it was apparent that questions about strategic positioning and withdrawal could be interpreted as implying particular body parts for either or both the participant or their sexual partners. The questionnaire is primarily paper-based, and unlike an online questionnaire, survey routing cannot be used to easily modify the wording of these items based on the respondent’s earlier responses. Given that these risk reduction strategies have become less important to report on for the research team in the context of PrEP and undetectable viral load, the team decided to remove these questions in response to this feedback. While this helped reduce the number of assumptions being made about bodies, there remains an ongoing tension about how in-depth and precise the measurement of sexual practices can or should be in surveys of this kind.

The insights in our study share similarities to those offered by Mooney-Somers and colleagues’ [16] when reflecting on their experiences in adapting a 25-year old survey for lesbian, bisexual, and queer women to better engage with and represent gender diverse participants. For example, the authors suggested that “survey questions will always contain compromises, but we must be open about the compromises we make, and commit to revisiting them.” [16], p. 241] In our case, some trans participants expressed critical views about how extensive the GCPS revisions were in terms of asking about specific body parts and practices. However, the amount of detail preferred by some community members is rarely feasible in a short paper-based survey, and particularly one being completed in a public setting where multiple, specific questions about body parts may be experienced as intrusive. A compromise of some kind is always necessary in balancing the objectives of the study (e.g., producing a cross-sectional quantitative analysis of HIV risk) and community expectations (e.g., a more comprehensive risk-based assessment suitable to a clinical context, or in-depth detail more suitable to qualitative studies). The research team invests in ongoing community engagement and partner consultation processes to ensure that the survey can maintain this balance over time, and ensure trust, community acceptability, and participation are prioritised alongside the research objectives of the study.

Mooney-Somers and colleagues [16] have also suggested that it is not possible to ‘future-proof’ questions about gender diversity because the way gender and sexuality are understood and lived continue to change, and priorities in how experience and identity come to matter in people’s lives also shift over time [33]. The approach taken for revising the GCPS therefore may not be an exact model for other studies, because understandings of sex, gender, sexuality, and sexual practice (and the language used to describe these) also differ markedly between contexts, and may be understood quite differently within ‘subcommunities’ relevant to a study. This study affirms these principles; although there are tips for inclusive survey design that may be transferable to other contexts and studies. The most important value of this study is to confirm the principles of community consultation, a willingness to adapt, and to be reflexive about survey methodology [34]. The process we followed could be adapted by others and could consider including other steps or methods that we did not use, including the use of focus group discussions, co-design workshops, or longitudinal cognitive interviews (e.g., inviting the same participants to do a repeat interview with a revised version of the questionnaire).

Anecdotal feedback from the most recent rounds of the survey (2024-25) since the changes were implemented have indicated that the changes continue to be viewed positively by participants. The research team has adjusted how it reports results from the surveys to include new measures, such as expanded details on female and non-binary sex partners [35, 36]. National analyses are underway by the research team, including disaggregating participants based on whether they report sex primarily with male partners, partners of all genders, or primarily with women and non-binary people only. Preliminary analyses suggest that there are differences in levels of HIV/STI testing and HIV prevention related to the gender of participants’ partners. Over time, it will also be possible to conduct specific analyses of TGD participants across the national sample, although this may be limited due to comparatively low sample sizes and statistical power. At the time of writing this article, these survey changes we have introduced have been followed by modest increases in the numbers of trans men, non-binary participants, and bi + participants in the surveys, with the majority of participants continuing to identify as cisgender gay men. This suggests that dedicated studies focused on bi + people and TGD are needed to provide further depth and understanding about HIV-related behaviour in these groups. However, we believe that the changes we have made to our study ensure that the study remains acceptable and relevant to communities we work with, with the potential to identify new trends affecting GBQ + men and non-binary people who have sex with men.

### Limitations and Strengths

A known limitation of cognitive interviewing is that the process “may lead to more carefully thought-out answers than a survey respondent might typically provide.” [23], p.354] To mitigate this, our analysis considered the proportion of participants who found the questions easy or non-problematic, and explored whether participants were able to answer questions (and appreciated their intended purpose) even if they provided critical feedback about wording. Further, most field interview participants expressed a consistent view of the overall acceptability of the revised survey. Although the field interviews were less in-depth, their immediate feedback following survey completion captured unprepared and unrehearsed participant sentiments and opinions about the survey, providing useful insight into participant perspectives about the new questionnaire. A strength of this study was combining insights from both methods together to evaluate the acceptability of the survey. Additionally, the overall consultation process ensured there were many opportunities to review the survey questionnaire and to ensure that it was acceptable to a broad range of stakeholders, including the representatives of community-based organisations from across Australia.

## Conclusion

Improving the design of questions and recruitment material to enable participants to more accurately share their demographic details and answer questions is important for improving the quality of data, but also willingness to participate and complete survey questions. Balancing the affirmation of community experience and expectations with the creation of a useable, reliable survey instrument is challenging and involves compromises. This process of consultation and revision of survey questions could be adapted in other surveillance systems to improve gender and sexuality inclusivity, and therefore the quality of the HIV and sexual health data collected in those systems.

## Data Availability

Research data are not shared.
